# Real-Time Functional Magnetic Resonance Imaging Dyadic Neurofeedback for Emotion Regulation: A Proof-of-Concept Study

**DOI:** 10.3389/fnhum.2022.910951

**Published:** 2022-05-26

**Authors:** Kara L. Kerr, Erin L. Ratliff, Zsofia P. Cohen, Stormie Fuller, Kelly T. Cosgrove, Danielle C. DeVille, Masaya Misaki, Amanda Sheffield Morris, Jerzy Bodurka

**Affiliations:** ^1^Department of Psychology, Oklahoma State University, Stillwater, OK, United States; ^2^Oklahoma State University Biomedical Imaging Center, Tulsa, OK, United States; ^3^Department of Pharmacology and Physiology, Center for Health Sciences, Oklahoma State University, Tulsa, OK, United States; ^4^Department of Behavioral Pediatrics, University of Oklahoma Health Sciences Center, Oklahoma City, OK, United States; ^5^Laureate Institute for Brain Research, Tulsa, OK, United States; ^6^Department of Psychology, The University of Tulsa, Tulsa, OK, United States; ^7^Department of Psychiatry, University of California, San Diego, San Diego, CA, United States; ^8^Stephenson School of Biomedical Engineering, The University of Oklahoma, Norman, OK, United States

**Keywords:** fMRI, neurofeedback, adolescence, emotion regulation, insula, parenting, dyad

## Abstract

Real-time fMRI (rt-fMRI) neurofeedback can be used to non-invasively modulate brain activity and has shown initial effectiveness in symptom reduction for psychiatric disorders. Neurofeedback paradigms often target the neurocircuitry underlying emotion regulation, as difficulties with emotion regulation are common across many psychiatric conditions. Adolescence is a key period for the development of emotion regulation, with the parent-adolescent relationship providing an important context for learning how to modulate one’s emotions. Here, we present evidence for a novel extension of rt-fMRI neurofeedback wherein a second person (the parent) views neurofeedback from the focal participant (adolescent) and attempts to regulate the other person’s brain activity. In this proof-of-concept study, mother-adolescent dyads (*n* = 6; all female) participated in a dyadic neurofeedback protocol, during which they communicated *via* active noise-canceling microphones and headphones. During the scan, adolescents described current emotionally upsetting situations in their lives, and their mothers responded while viewing neurofeedback from the adolescent’s right anterior insular cortex (aIC)—a key hub for emotion-related processing. The mother was instructed to supportively respond to her daughter’s negative emotions and attempt to downregulate the aIC activity. Mean right aIC activation during each run was calculated for each adolescent participant, and results revealed a downward trend across the session (β = −0.17, *SE*_β_ = 0.19, Cohen’s *f*^2^ = 0.03). Results of this proof-of-concept study support further research using dyadic neurofeedback to target emotion-related processing. Future applications may include therapist-client dyads and continued research with parents and children.

**Clinical Trial Registration:**

[www.ClinicalTrials.gov], identifier [NCT03929263].

## Introduction

Adolescent mental health diagnoses have been increasing at an alarming rate over the past several decades (Collishaw et al., 2004; Keyes et al., 2019; Mojtabai and Olfson, 2020). Alongside these already increasing rates, pivotal events in recent years such as the COVID-19 pandemic and social and political unrest are likely to have negative effects on the mental health of youth globally. An estimated half of all psychiatric disorders develop before 18 years of age and contribute to disease burden throughout life (Kessler et al., 2010; Jones, 2013). Mental health conditions are now one of the top 10 causes of disability globally and cost an estimated $50 billion in the U.S. alone (Baxter et al., 2014), highlighting the need for prevention and intervention efforts to mitigate this growing public health crisis.

Despite decades of research demonstrating the efficacy and effectiveness of psychotherapy (e.g., Das et al., 2016; Krebs et al., 2018), barriers to treatment (e.g., systemic and structural barriers, stigma) and lacking mental health literacy are pervasive (Waid and Kelly, 2020). Nearly half of children and youth with psychiatric disorders do not receive any mental health services (Whitney and Peterson, 2019). Of the individuals who do receive treatment, lifetime rates of recurrence are extremely high, particularly for internalizing disorders such as anxiety and depression. For example, at least half of adolescents treated for major depressive disorder experience another episode within 2 years (Curry et al., 2011). Novel interventions such as brain modulation approaches could help to address the growing mental health crisis among youth.

Psychopathology often emerges during adolescence as emotion regulation (the ability to recognize and modulate one’s emotions) and related brain structures are continuing to develop (Vink et al., 2014; Zimmermann and Iwanski, 2014; Silvers, 2022). Adolescents who experience difficulties in emotion regulation are at a heightened risk for mental health disorders (Silk et al., 2003; Gonçalves et al., 2019), particularly internalizing disorders such as depression, anxiety, and suicidality. This risk is particularly pronounced in adolescent females, who experience these disorders at nearly three times the rate of their male counterparts (Breslau et al., 2017). In addition to internalizing disorders, emotion dysregulation is related to other psychiatric conditions including substance use disorders (Wilcox et al., 2016), oppositional defiant disorder (Cavanagh et al., 2017), and borderline personality disorder (Gratz et al., 2016). Behavioral therapies targeting emotion regulation have shown efficacy across psychiatric diagnoses (Khakpoor et al., 2019; Sakiris and Berle, 2019). As many of these disorders onset during adolescence, novel neuroscience-based interventions have the potential to aid in the prevention and treatment of many different psychiatric conditions.

The tripartite model of emotion regulation development (Morris et al., 2007) posits that emotion regulation skills develop through observation of parents’ own regulation strategies, parent emotion socialization practices, and the family emotional climate. Numerous studies have documented associations between emotion-related parenting and adolescent emotion regulation development, supporting the notion that the development of emotion regulation occurs within the context of the family and, more specifically, the parent-child relationship (Morris et al., 2018). For example, research suggests supportive parent emotion socialization practices, including emotional responsiveness, open communication, and problem-solving, contribute to the development of effective adolescent emotion regulation skills which, in turn, may promote positive adolescent development and protect against psychopathology (Brenning et al., 2015; Perry et al., 2020). In contrast, unsupportive parent emotion socialization practices (e.g., invalidating, critical, or punitive reactions to adolescent emotional distress) have been shown to undermine optimal development of emotion regulation, which may influence the development of internalizing and externalizing disorders (Cui et al., 2020; Byrd et al., 2022). During adolescence, parent-adolescent relationships become more egalitarian (Lougheed, 2019), and parents often engage in less supportive emotion socialization strategies, likely reflecting expectations for greater self-regulation (Klimes-Dougan et al., 2007). However, less supportive emotion socialization strategies have been linked to high levels of adolescent emotion dysregulation (Byrd et al., 2022), suggesting that despite the transformations that occur in the parent-adolescent relationship, emotional support is still critical. Interventions that target parents’ emotion socialization and positive parenting can therefore affect their adolescents’ emotion regulation and protect against internalizing disorders (Kehoe et al., 2014).

Neuroimaging studies suggest normative variations in parenting practices can influence adolescent brain structure and function, specifically in regions involved in emotion processing and regulation (for an in-depth review, see Tan et al., 2020). For example, higher levels of supportive parenting practices are associated with smaller amygdala volumes (Whittle et al., 2014), larger hippocampal volumes (Luby et al., 2016), and increased cortical thinning in the orbitofrontal cortex in adolescents (Whittle et al., 2014). Regarding brain function, Romund et al. (2016) found supportive parenting practices were related to lower amygdala activation in adolescents when viewing negative emotional stimuli. In contrast, unsupportive or negative maternal practices during a parent-child interaction were shown to be related to increased amygdala activation in response to negative emotional stimuli (Pozzi et al., 2020).

The anterior insular cortex (aIC) is particularly important in the development of emotion regulation throughout adolescence. The aIC links cortical and subcortical regions of the brain (Shaw et al., 2008; Smith et al., 2014) and is critically important in decision-making as it integrates cognitive and emotional processes. Further, parent negative statements made during a parent-adolescent conflict discussion and parents’ own anxiety symptoms were both associated with increased adolescent aIC activation during a dyadic error processing task (Cosgrove et al., 2019, 2022), and hyperactivation of the aIC is associated with depression and a history of childhood maltreatment in adolescents (McLaughlin et al., 2015; Miller et al., 2015). A previous study targeting the aIC for real-time functional magnetic resonance imaging (rt-fMRI) neurofeedback in adolescents found that downregulation of the aIC was associated with top-down information flow (effective connectivity) from cortical structures to the amygdala (Cohen Kadosh et al., 2016), likely influencing the emotion regulation network in the brain. Downregulation of the aIC in the context of emotional distress may thus serve to promote healthy emotion regulation in adolescents and protect against the development of internalizing symptoms.

Given that brain plasticity is particularly high during adolescence (Laube et al., 2020) and that emotion regulation is a critical element in both internalizing and externalizing psychopathology (Heleniak et al., 2016; Compas et al., 2017) and is highly influenced by the parent-child relationship (Morris et al., 2007), parent modulation of adolescents’ aIC activity presents an opportunity for non-invasive neuromodulation of cognitive and affective states. This neuromodulation may have long-term positive outcomes for adolescent emotion regulation development and mental health. In the present proof-of-concept study, we use non-invasive rt-fMRI neurofeedback to target dyadic emotion regulation processes between mothers and their adolescent daughters. Rather than individual feedback, the neurofeedback from the adolescent’s aIC is presented to her mother (i.e., dyadic neurofeedback; rt-fMRI-DNF) during an emotion discussion task. This study included one rt-fMRI-DNF session for each dyad, as prior research has found long-term effects on the use of effective emotion regulation strategies in daily life following a single rt-fMRI neurofeedback session (MacDuffie et al., 2018). By targeting a central hub of the emotion regulation network and capitalizing on parent-child dyadic relationships, this proof-of-concept study applies a novel approach to enhancing and promoting adaptive emotion regulation in adolescents.

## Materials and Methods

### Participants

Ten adolescent females (age range for inclusion: 13–16 years) and their biological mothers participated in this proof-of-concept study. This study only included adolescent females due to their higher risk for disorders related to emotion dysregulation, such as depression (Breslau et al., 2017). Participants were recruited from the community primarily through electronic flyers distributed through local public schools. Study procedures were approved by the Oklahoma State University Center for Health Sciences Institutional Review Board (IRB protocol #2017011). Parent participants provided written informed consent for their own and their daughters’ participation. Adolescent participants provided written informed assent. Informed consent procedures were conducted in accordance with the Declaration of Helsinki. Parent and adolescent participants each received financial compensation for their participation in the study.

### Procedures and Measures

Participants completed an initial research session during which the Mini International Neuropsychiatric Interview (MINI 7.0, Sheehan et al., 1997; MINI KID 7.0, Sheehan et al., 2010) was administered separately to the parent and adolescent to screen for psychiatric disorders. Participants also completed survey measures and magnetic resonance imaging (MRI) safety screening. In order to first test rt-fMRI-DNF in a psychiatrically healthy sample, dyads were excluded from participation in the neurofeedback protocol if either participant met criteria for a current psychiatric disorder. Dyads were also excluded if the adolescent had a history of a psychiatric disorder. As both the mother and daughter participated in fMRI scanning as part of a larger study, dyads were additionally excluded for left-hand dominance (as assessed by the Edinburgh Handedness Inventory; Oldfield, 1971), current pregnancy or breast feeding, psychotropic medication use within the past 3 weeks (6 weeks for fluoxetine), or general fMRI contraindications (e.g., ferrous metal implants) in either participant. The initial screening session was originally conducted in person at the Laureate Institute for Brain Research (LIBR; *n* = 4 dyads), but after the onset of the COVID-19 pandemic we began conducting this initial session virtually in order to reduce risk for research participants and staff (*n* = 6 dyads). For the virtual visits, consent forms were signed electronically in REDCap at the beginning of the session. Informed consent discussions and clinical interviews were conducted *via* Zoom, and participants completed survey measures online following the session.

Dyads meeting inclusion criteria were invited to complete the neurofeedback session at LIBR. Participants completing the scanning session during the COVID-19 pandemic first completed a temperature check, symptom screening, and a rapid COVID-19 test upon arrival to the session. All participants were screened for recent drug or alcohol use and current pregnancy. Participants were given instructions for the Emotion Discussion Task (described below) and practiced lying still in a mock scanner environment.

### Emotion Discussion Task and Dyadic Neurofeedback

Participants completed the Emotion Discussion Task (adapted from Suveg et al., 2005) while undergoing fMRI hyperscanning (i.e., simultaneous scanning of the parent and adolescent in two identical scanners). Parent fMRI data were collected to examine associations between parenting behaviors and brain activation (data not presented here due to the small sample size). During the Emotion Discussion Task, the adolescent and her mother take turns speaking in 40 s blocks. For each of five scanning runs, the adolescent discusses a different upsetting emotion situation (e.g., “My friends didn’t invite me to go to the movies with them”), and the mother responds. During the neurofeedback runs (runs 2, 3, and 4), the mother views a moving bar representing her daughter’s brain activity and attempts to downregulate the bar by what she says to her daughter. No neurofeedback is presented in the first run in order to obtain an estimate of the adolescents’ aIC activity while listening to her mother without neurofeedback. No neurofeedback is presented during the final run in order to determine if the effects of neurofeedback training persist without neurofeedback being shown. Additional details of the task are given below. No control condition or group was included for this proof-of-concept study, as the primary aims were to test the fMRI task protocol and determine if a second person could regulate the focal participant’s aIC activity.

Prior to the scan, parents and adolescents were given task instructions separately, and adolescents were asked to think of five recent upsetting situations (one for each scanning run) and to provide brief descriptions and an emotional rating (“How upset are you when you think about this (0–10)?”) for each. Parent participants were informed that their daughters would be telling them about current emotional situations in their lives and that they would be asked to respond supportively. Specifically, they were instructed, “During these trials, respond to your daughter as you typically would if she came to you to talk through a problematic situation.” They were also instructed that during neurofeedback trials they should use the moving red bar (visual representation of aIC activity) as feedback on their responses, with the goal of lowering the bar (i.e., downregulating their daughter’s aIC activation). Parent participants were informed that their goal should be to reduce the intensity of their daughters’ negative emotions, and that doing so should lower the level of the red bar. They were instructed to attempt to match the level represented by a stationary blue bar presented next to the red bar. Finally, parents were encouraged not to focus too much on the red bar or get discouraged if it remained high.

Participants communicated during the task using active noise-canceling microphones and headphones (OptoActive II NC Microphones and ANC Headphones, Opto Acoustics Ltd.). Prior to the Emotion Discussion Task runs, a 1 min sound check was performed during which the parent and adolescent conversed over the headsets while an echo-planar imaging (EPI) scan was acquired to ensure the sound system was working properly and to identify any adjustments (e.g., microphone placement) that needed to be made prior to the Emotion Discussion Task scans. No neurofeedback was presented during the first (baseline) and last (transfer) runs in order to determine baseline aIC activity and the persistence of effects in the absence of neurofeedback, respectively.

**FIGURE 1:**
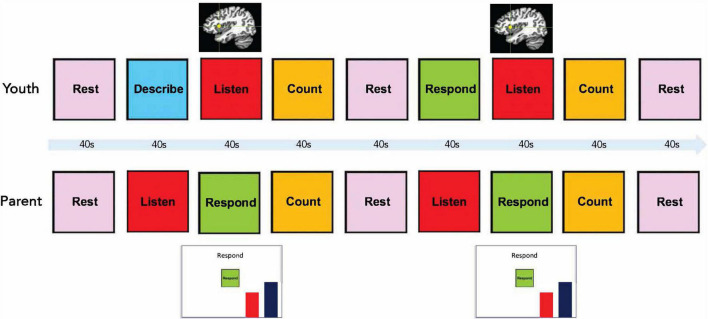
Emotion discussion task design. Each block had a duration of 40 s. During Listen blocks for the adolescent, the mother spoke to the adolescent *via* an active noise-canceling headset while viewing a moving red bar representing neurofeedback from her daughter’s right anterior insula.

The Emotion Discussion Task consisted of four conditions presented in 40 s blocks: Rest, Count, Listen, and Describe/Respond (Figure 1). Participants were cued for each block by text on a screen. During the first Describe block, the adolescent described the emotional situation. A different emotional situation was selected for each scanning run. The mean emotion rating for all five situations was calculated, and the two situations with ratings closest to the mean were assigned the baseline and transfer runs. The remaining three situations were presented in a random order during the neurofeedback runs. An experimenter verbally informed the participant prior to each run regarding which situation was to be discussed (e.g., “During this run, we would like you to discuss the argument you had with your friend”). The first Describe block in each run was followed by a Listen/Respond block, during which the adolescent was instructed to listen while her mother responded to the situation she had just described. During these blocks for neurofeedback runs, the mother simultaneously viewed a vertical red bar reflecting her daughter’s aIC activity. These blocks were followed by a Count block, during which participants were instructed to mentally count backward from 300 by a number specified on the screen (for example, by 6: 300, 294, 288…). The Count blocks were included in order to bring the adolescent’s aIC activity back to baseline and were followed by a 40 s Rest block. This was followed by a Respond block, during which the adolescent responded to what her mother had said previously (no neurofeedback was presented to either participant). The mother then had another Respond block with neurofeedback, followed by another Count and Rest block (total run time = 6 min). Each participant was cued with the word “Listen” while the other participant was speaking.

Following each scanning run, parent participants were asked to separately rate from 1 (“Not at all”) to 10 (“Extremely”) how effective they think they were at reducing their daughter’s emotions and how effective they think they were at lowering the red bar (neurofeedback trials only). Adolescents were asked to rate on the same scale how upset they currently feel. Participants reported their ratings verbally to the experimenter, who logged their responses.

### MRI Data Acquisition

Functional and structural brain images were acquired using a General Electric Discovery MR750 whole-body 3 Tesla MRI scanner. A system-provided receive-only 8-element surface head coil was used for MR signal reception. The scanner was equipped with real-time motion monitoring using AFNI. A T1-weighted MRI scan with magnetization-prepared rapid gradient echo (MPRAGE) sequence with SENSE was acquired for use as an anatomical reference for the fMRI analyses. The MPRAGE sequence employed the following parameters: FOV/slice = 240/1.2 mm, 120 axial slices per volume, image matrix = 256 × 256, voxel volume = 0.94 × 0.94 × 1.2 mm^3^, TR/TE = 5/1.94 ms, SENSE acceleration factor *R* = 2, flip angle = 8°, inversion time = 725 ms, sampling bandwidth = 31.25 kHz, scan time = 4 min 59 s.

Functional scans were acquired using a single-shot gradient-recalled EPI sequence with sensitivity encoding (SENSE) and the following EPI parameters: FOV = 240 mm, slice thickness = 2.9 mm, 40 axial slices per volume, acquisition matrix = 96 × 96, repetition/echo time (TR/TE) = 2,000/25 ms, SENSE acceleration factor *R* = 2, flip angle = 90°;, sampling bandwidth = 250 kHz, number of volumes = 180. EPI images were reconstructed into a 128 × 128 matrix, with an fMRI voxel volume of 1.875 mm × 1.875 mm × 3.4 mm.

### Online Data Analyses

All imaging analyses were performed using AFNI.^1^ Real-time neurofeedback was implemented using custom software. An automated script generated an anatomically defined 5 mm spherical region-of-interest (ROI) centered at the right aIC (locus of 38, 16, 3 in Talairach space; Talairach and Tournoux, 1988). Prior to the neurofeedback runs, a short (20 s) EPI scan was acquired. The participant’s MPRAGE image was transformed into Talairach space. The aIC ROI was transformed to the participant’s original MPRAGE space and then to the EPI space as defined from the short EPI scan. During neurofeedback runs, the acquired EPI volumes were volume registered to the same EPI volume as the ROI using the AFNI real-time plug-in. This plug-in was also used to export the mean value for the aIC ROI (percent signal change relative to average fMRI signal during preceding Rest block) for display to the participant. The moving bar displaying the aIC neurofeedback signal was updated every 2 s. The bar height at each time point represented the moving average of the current and previous two fMRI percent signal change values in order to reduce spurious fluctuations due to noise in the signal. A stationary blue bar was also presented next to the red bar as a visual goal for lowering the red bar (Figure 1).

### Offline Data Analyses

Each participant’s anatomical scan was first non-linearly warped to Talairach space using the AFNI program @SSWarper. The remaining preprocessing steps were then performed using afni_proc.py. The first three EPI volumes were removed to allow the signal to reach steady state. A single image transformation implemented both spatial normalization and motion correction. The EPI images were resampled to a 1.75 mm^3^ grid. A 6 mm full-width at half-maximum Gaussian kernel was used for spatial smoothing. Preprocessing also included RETROICOR (Glover et al., 2000) and respiration per volume time correction (Birn et al., 2008). The signal was scaled to represent percent signal change relative to the mean of each voxel. The general linear model analysis included regressors for the four active conditions (Describe, Listen, Count, Respond) as a boxcar function convolved with a hemodynamic response function and motion parameters and derivatives. Individual time points were censored if more than 5% of the voxels were considered outliers based on 3dToutcount’s “automask” function or if their Euclidean-normalized motion derivative exceeded 0.3. Each scanning run was processed individually to assess changes in aIC activity across the scanning session. Participants with excessive motion across scanning runs (censor fraction during Listen [neurofeedback] condition > 15%) were excluded from data analysis (*n* = 4; resulting in a final sample size of 6 dyads). Data from a single run (second neurofeedback training run) were also excluded from one participant due to excessive motion in that run only. Another participant’s final transfer run was not included in analyses due to difficulties with the sound system that did not allow the dyad to interact during that run. Data were also visually inspected for quality control to ensure proper alignment and no irregularities in preprocessing.

### Statistical Analyses

Due to the small sample size for this proof-of-concept study, statistical analyses are focused on effect sizes rather than statistical significance. The AFNI program 3dROIstats was used to obtain participants’ means during the Listen condition for each run. Beta values from the GLM for the Listen condition were averaged within a 5 mm aIC mask centered at 38, 16, 3 (Talairach space). To assess for a linear trend in aIC activity across scanning runs, a least squares linear regression was run in R (R Core Team, 2019) with scanning run number (1–5) as the predictor and right aIC activity as the dependent variable.

To assess whether participants rated their emotions as less negative following DNF, Cohen’s *d* was used with participants’ mean ratings of how upset they were following each scanning run compared to their ratings for each situation prior to the scan.

## Results

**FIGURE 2:**
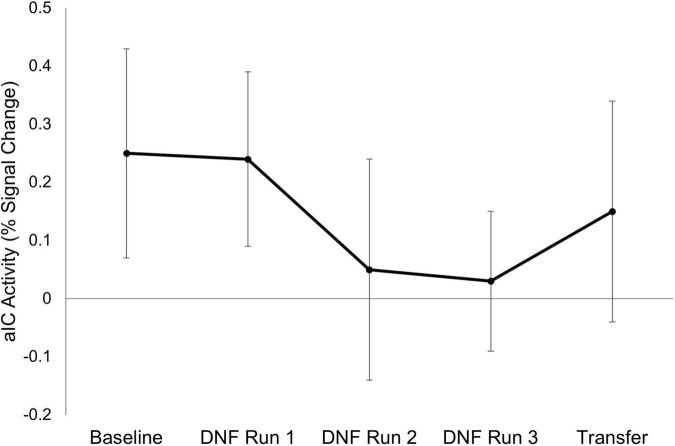
Right anterior insula activity across scanning runs. Mean activation in adolescents’ right anterior insular cortex (aIC) is shown for each scanning run (*n* = 6). Error bars represent the standard error of the mean. Neurofeedback was not presented during the Baseline and Transfer runs.

Demographic information for the final sample (*n* = 6) is presented in Table 1. Results of the neuroimaging analyses revealed a small effect for a downward trend for right aIC activity across the session (β = −0.17, *SE*_β_ = 0.19, Cohen’s *f*^2^ = 0.03; Figure 2). There was also a small effect size (Cohen’s *d* = −0.22) for decreased right aIC activity in the transfer run (aIC percent signal change *M* = 0.15, *SD* = 0.43) as compared to the baseline run (aIC percent signal change *M* = 0.25, *SD* = 0.45), indicating the learned effects persisted without the parents relying on the neurofeedback signal.

**TABLE 1:** Sample demographics.

	Youth	Parents
**Race (*n*)**		
White, non-Hispanic	5	6
More than one race	1	0
**Household income (*n*)**		
50,000–$75,000		3
100,000 or greater		3
**Education (*n*)**		
High school graduate/GED		1
Some college or trade school		3
College degree		2
Age in years (*M* [*SD*)]	15.33 (1.21)	43.00 (4.60)

Adolescent participants’ negative emotion ratings (0–10 rating of how upset they felt) were also lower (Cohen’s *d* = −3.69) after the neurofeedback runs (*M* = 2.11, *SD* = 1.46) as compared to their ratings of each situation prior to the scan (*M* = 6.43, *SD* = 0.79). Additionally, the mean pre-scan rating of 6.43 in response to “How upset are you when you think about this situation?” indicates that participants were able to identify five moderately upsetting situations to discuss during the scan, providing evidence for the feasibility of the experimental paradigm in this population. Parents also felt that they were able to effectively modulate their daughters’ aIC activity (*M* = 6.67, *SD* = 2.12 on the 0–10 scale with 10 being “extremely” effective) and emotions (*M* = 5.96, *SD* = 2.45).

## Discussion

This is the first study to our knowledge to demonstrate the feasibility of rt-fMRI-DNF, representing an important advancement in the use of non-invasive brain modulation approaches. DNF can broaden the populations and targets for brain modulation, with potential benefits for both individual health and relationship quality. The current study tested this approach in the context of a highly impactful and emotional relationship, that of the parent and child. Our ecologically valid approach demonstrated how a second person can modulate a focal participant’s aIC activity through emotionally supportive statements, with implications for both our understanding of the brain and the development of interventions.

Using a novel emotion discussion paradigm, we found preliminary evidence that mothers were able to downregulate their daughters’ aIC activity. This was primarily demonstrated through a consistent, downward trend in aIC activity across time over the course of the scanning session. Results also showed that the decreased activation was maintained during the transfer run, suggesting that mothers may be able to apply what was learned from the neurofeedback training to future interactions with their daughters without the need for reliance on a neurofeedback signal. Emotion ratings from the adolescent participants also supported the possible utility of this approach, as adolescents’ report of their own negative emotions were lower following neurofeedback than they were when asked to think about each emotional situation prior to the scan. It is possible that these effects could build over the long term, with increased positive, validating interactions between adolescents and their parents leading to better emotion regulation and protection against the development of internalizing symptoms.

The aIC has been a target of many past rt-fMRI neurofeedback studies of emotion regulation (Caria et al., 2007, 2010; Veit et al., 2012; Berman et al., 2013; Ruiz et al., 2013; Lawrence et al., 2014; Sitaram et al., 2014; Buyukturkoglu et al., 2015; Zilverstand et al., 2015; Yao et al., 2016), including a study of adolescents (Cohen Kadosh et al., 2016). In the study by Cohen Kadosh et al. (2016), adolescents’ self-regulation of aIC activity influenced effective connectivity in the neural network underlying emotion regulation, including connectivity between the amygdala, aIC, and cortical structures such as the dorsolateral prefrontal cortex. In adults diagnosed with specific phobia, successful downregulation of the aIC using rt-fMRI neurofeedback was related to reduced anxiety at a 3-month follow-up (Zilverstand et al., 2015), again indicating that successful aIC regulation may have important implications for emotion regulation and mental health. Difficulties in emotion regulation represent a core vulnerability to both internalizing and externalizing disorders across the lifespan, and thus enhancing adaptive emotion regulation development in children and adolescents may protect against the onset of these disorders. As parents continue to influence adolescents’ emotion regulation and related neurobiological development into the teen years (Tan et al., 2020), using neurofeedback to assist parents in their interactions with their adolescents could be a key target in promoting positive socioemotional development.

The primary limitations of the current study are the small sample size and lack of control condition. As a proof-of-concept study, we sought to test the experimental paradigm in a small sample to inform a larger, more comprehensive trial in the future. A larger sample would enable the examination of how pre-existing individual differences in emotion regulation skills in both the parents and adolescents may affect DNF outcomes. A significant portion of our sample was also excluded due to excessive motion. While this is common for fMRI research with children and adolescents, more extensive training protocols with participants and/or an older sample may be needed for future neurofeedback studies. Separating the speaking blocks by a short rest block may also aid to help eliminate residual motion from the adolescent’s speaking turn. Additionally, while participants’ aIC activity decreased across scanning runs, we cannot determine if this was an effect of DNF due to the lack of a control condition. Another limitation is that our sample only included female participants and biological mothers. Future studies should include other genders and caregiver roles (e.g., adoptive parents) as well as a more diverse, representative sample in terms of socioeconomic status and race and ethnicity to determine the generalizability of rt-fMRI-DNF for the broader population.

There are a wide variety of future directions and potential applications for rt-fMRI-DNF. This method needs to be tested in a larger sample with a control condition (e.g., parenting without neurofeedback; sham feedback) for a more rigorous evaluation of its efficacy in downregulating the aIC. A larger sample would also allow for whole-brain and connectivity analyses to determine what regions may be affected in addition to the aIC. Measures of emotion regulation and internalizing symptoms are needed to determine if the effects on the brain are reflected in emotions and behavior. Long-term follow-up assessments may be needed, as rt-fMRI-DNF’s effects on the developing brain may not become evident behaviorally for months or years. An assessment of which parenting behaviors tend to be most effective in reducing aIC activation would also be useful in developing interventions for use outside the scanner. Dyadic neurofeedback could also be combined with parenting programs (e.g., Tuning into Teens, Kehoe et al., 2014) to possibly enhance the effectiveness of such interventions. Dyadic neurofeedback also has many potential applications outside of the parent-adolescent relationship. Parents and younger children, romantic couples, therapists and clients, workplace teams, and many other types of relationships may benefit from this approach. Additionally, hyperscanning methods would allow the opportunity to modulate not just individual brain activation but also interbrain synchrony as dyads are interacting (Gvirts Provolovski and Perlmutter, 2021; Saul et al., 2022). Dyadic neurofeedback broadens our focus from the individual to their social relationships and harnesses these relationships for the promotion of overall wellbeing.

While preliminary, the current findings provide initial evidence for the feasibility of a two-person, rt-fMRI-DNF approach for modulating activity of the aIC, a key hub in the emotion regulation network. Non-invasive brain modulation approaches targeting regions and networks underlying emotion-related processes have great potential to advance efforts in understanding, treating, and preventing psychiatric disorders. The use of such approaches during childhood and adolescence may be of particular benefit, as the brain is still developing and more responsive to environment and experience. For these same reasons, however, research must proceed cautiously, with care taken to ensure there are no iatrogenic effects. Future directions for this work include testing this protocol in a larger sample and applying these methods to other significant relationships.

## Data Availability Statement

The raw data supporting the conclusions of this article will be made available by the authors, without undue reservation.

## Ethics Statement

The studies involving human participants were reviewed and approved by the Oklahoma State University Center for Health Sciences Institutional Review Board. Written informed consent to participate in this study was provided by the participants’ legal guardian/next of kin.

## Author Contributions

KK, ER, KC, DD, MM, AM, and JB contributed to the conception and design of the study. JB and MM designed and developed the software used for neurofeedback. KK, ER, and ZC wrote the first draft of the manuscript. KK, SF, ER, KC, and DD contributed to data collection. KK and SF contributed to project administration and data curation. With the exception of JB (posthumous authorship due to significant contributions to study conception and design), all authors contributed to manuscript revision and read and approved the submitted version.

## Conflict of Interest

The authors declare that the research was conducted in the absence of any commercial or financial relationships that could be construed as a potential conflict of interest.

## Publisher’s Note

All claims expressed in this article are solely those of the authors and do not necessarily represent those of their affiliated organizations, or those of the publisher, the editors and the reviewers. Any product that may be evaluated in this article, or claim that may be made by its manufacturer, is not guaranteed or endorsed by the publisher.

## Footnotes

1
https://afni.nimh.nih.gov/
